# Coherent quantum transport features in carbon superlattice structures

**DOI:** 10.1038/srep35526

**Published:** 2016-10-19

**Authors:** R. McIntosh, S. J. Henley, S. R. P. Silva, S. Bhattacharyya

**Affiliations:** 1 Nano-scale Transport Physics Laboratory, School of Physics, and Centre of Excellence in Strong Materials, University of the Witwatersrand, Private Bag 3, WITS 2050, Johannesburg, South Africa; 2 Advanced Technology Institute, University of Surrey, Guildford, Surrey, GU2 7XH, UK

## Abstract

Whilst resonant transmission is well understood and can be fully harnessed for crystalline superlattices, a complete picture has not yet emerged for disordered superlattices. It has proven difficult to tune resonant transmission in disordered diamond-like carbon (DLC) superlattices as conventional models are not equipped to incorporate significant structural disorder. In this work, we present concurrent experimental and theoretical analysis which addresses resonant transmission in DLC superlattices. Devices were fabricated by growing alternate layers of DLC with different percentages of *sp*^3^ hybridized carbon.Coherent quantum transport effects were demonstrated in these structurally disordered DLC superlattices through distinct current modulation with negative differential resistance (NDR) in the current-voltage (I-V) measurements. A model was developed using tight-binding calculations assuming a random variation of the hopping integral to simulate structural (bond-length) disorder. Calculations of the I-V characteristics compliment the interpretation of the measurements and illustrate that while DLC superlattice structures are unlike their classical counterparts, the near-field structural order will help with the confinement of quantised states. The present model provides an empirical guide for tailoring the properties of future devices, giving rise to much hope that carbon electronics operating at high frequencies over large areas can now be developed.

Amorphous superlattices, known to be advantageous in overcoming the lattice mismatch problems of crystalline semiconductors, have long been studied towards developing high-speed oscillators and detectors^1^. However while the physics of resonant transmission is well known for crystalline materials, disorder complicates the conventional concept of resonance in amorphous superlattices as the eigenenergies are no longer well defined. Yet, localized states as found in DLC, already confine electrons, and so move up in energy any eigenstates^2^. This will decrease trapping. Pushing the limits of device miniaturization, molecular electronic devices have generated significant interest^3^,^4^. Of particular interest is the origin of the negative differential resistance found in these systems which is still not fully understood^3^,^5^,^6^ due to the inherent uncertainty in isolating the effects of Coulomb interactions between charge carriers, the electron-phonon interaction and quantum interference effects. Electron-phonon interactions also play a key role in designing electronic devices in 2D materials such as graphene, and so understanding interfaces in superlattices is key to novel phenomena and their applications^7^.

Bridging these fields are amorphous carbon (a-C) superlattices which are formed through alternating regions of DLC (predominantly *sp*^3^-*C*) and more graphitic carbon (with a much lower percentage of *sp*^3^-*C*). This is unlike conventional band-gap engineered superlattices which rely on dopants to modulate the band structure. In DLC superlattices, molecular chains and aromatic ring clusters often form within the superlattice structure^8^,^9^. DLC superlattices display a wide range of attractive properties, such as high mechanical strength and wear resistance, high breakdown voltage^10^,^11^, excellent thermal conductivity and fast-switching capabilities^12^ along with bio-compatibility. DLC superlattice devices would facilitate a new class of devices with robust operating conditions. Quantum confinement has been firmly established in these disordered superlattice structures^13^ which showed evidence of negative differential resistance (NDR)^14^,^15^,^16^. DLC double barrier structures showed a greater peak to valley ratio as well as demonstrating high frequency performance capabilities up to 110 *GHz* and bias-related hysteresis in the current-voltage characteristics^12^. In addition, quantized conductance features were demonstrated which is strongly suggestive of filamentary (1D) channels through the superlattice^12^.

The role of bond aspect ratio and disorder on the transmission coefficient of disordered carbon superlattices has been addressed theoretically^17^,^18^ where the role of structural disorder was incorporated through a random variation of the hopping integral about a mean value. Resonant transmission was demonstrated despite the high degree of structural disorder. This model was extended to relate the changes in localization length with disorder in thin graphitic films grown by laser ablation^19^, successfully relating structural changes due to disorder to transport properties. Recently, this model was applied to interpret experimental measurements on the current-voltage characteristics of nitrogen incorporated a-C superlattices. This work illustrated the effects of carbon networks (chains) forming within the superlattice which results in significant bond-alternation in the *sp*^3^-*C* regions which was included in the simulations^11^.

Here we study the I-V characteristics of DLC superlattices at different temperatures. Negative differential resistance is demonstrated in superlattices with different microstructures. We interpret these effects using a 1*D* tight-binding model developed for structurally disordered superlattices with structural disorder (arising due to bond length disorder) included through a Gaussian distribution of the hopping integral about some mean value. Experimental features are interpreted in light of this model and by investigating different structural configurations. We illustrate that the barrier potential dominates over other factors (such as the barrier or well width, which play a more significant role in crystalline superlattices) and should be exploited in tuning the properties of future devices. We model the effects of nitrogen inclusion and bond alternation which are shown to enhance the electron confinement and result in improved resonant tunneling and consequently an increase in the peak-to-valley ratio of regions of NDR.

## Results

### Measured Transport Properties

**Figure 1:**
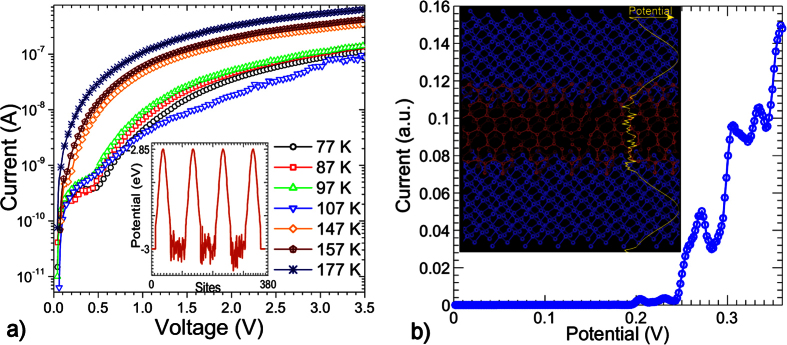
(**a**) Current-voltage characteristics of a DLC superlattice with 4 barriers of 7 nm and 3 well of 7 nm. Inset is the hopping term at each lattice site. (**b**) Calculated current-voltage characteristics for the same device configuration. Inset is a schematic diagram of the superlattice structure showing two *sp*^3^ hybridized barriers (regions with blue atoms) an *sp*^2^ hybridized quantum well (region with maroon atoms), with an illustration of the potential in each region overlaid in yellow.

Figure 1a shows the I-V characteristics of a DLC superlattice with 3 wells of width 7 *nm* and 4 barriers of the same width. There is a small region of current suppression at around 0.4 *V*, indeed showing slight negative differential resistance at low temperatures. The symmetry between the barrier and well width favors resonant transmission (see calculations later on). The temperature dependence is as expected for a quantum superlattice structure, showing no sign of NDR or current suppression when the temperature is increased such that inelastic scattering becomes significant while carrier confinement is reduced by emission processes such as Poole-Frenkel and Schottky emission.

**Figure 2:**
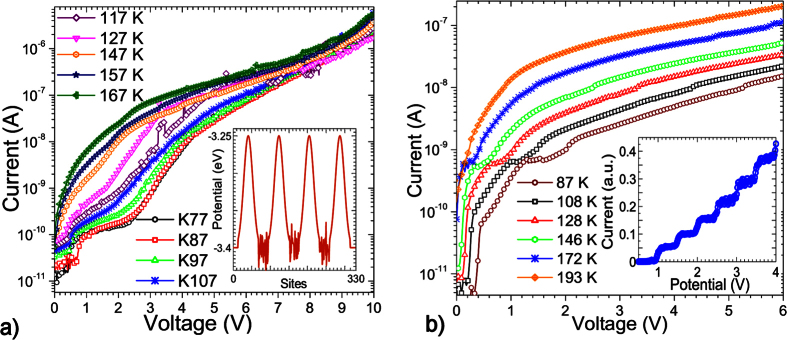
(**a**) Current-voltage characteristics of a DLC superlattice with 4 barriers of 7 nm and 3 wells of 4 nm. Inset is the hopping term at each lattice site. (**b**) Current-voltage characteristics of a DLC superlattice with 4 barriers of 8 nm and 3 wells of width 2 nm. Inset is the calculated current-voltage characteristics for the same device configuration.

Figure 2a shows the I-V characteristics of an amorphous carbon superlattice structure with 3 wells of width 4 *nm* and 4 barriers of width 7 *nm*. At low temperatures, there is strong suppression of the current until a sharp increase in the current at 0.8 *V* followed by prominent saturation of the current up to approximately 2.4 *V*. With increasing temperature, this feature shifts to lower potentials due to the increased thermal energy of charge carriers and broadening of the distribution of energies. This is indicative of resonant transmission despite the inherent disorder, pinholes and channels in the material. However there is insufficient confinement to result in negative differential resistance. Instead a wide range of resonant energies form a broad region of current suppression.

The I-V characteristics for a superlattice with 3 wells of width 2 *nm* and 4 barriers of width 8 *nm* are shown in Fig. 2b. At low temperatures, there are two clear step-like features. At 87 *K*, the first occurs at around 1.5 *V* while the second is found at around 2 *V*. There is also an additional step at high potentials (around 5.2 *V* at 87 *K*). The first step-like feature shows slight NDR which persists up to 146 *K*. This feature is less sensitive to temperature than the features in other devices, consistent with the enhanced confinement which would be expected for a thicker barrier. Despite the large difference between the widths of the quantum wells of this device and the previously discussed device, the resonant transport features are quite similar.

### Calculated Transport Properties

Figure 1b shows the I-V characteristics calculated for an a-C superlattice with four barriers with a width of 7 *nm* and three wells of the same width, to be compared with the device shown in Fig. 1 (the profile is shown in the inset of Fig. 1a). A schematic diagram of the disordered DLC superlattice is shown in the inset in Fig. 1b. The relatively ordered *sp*^3^ hybridized regions are represented with blue atoms. The schematic shows the inherent disorder in the *sp*^2^-*C* regions (represented with maroon atoms) where there is bond-length disorder as well as the inclusion of *sp*^3^ hybridized impurities and vacancies. The schematic also shows the mixture of *sp*^2^ and *sp*^3^ hybridization at the interface. This mixture is unique to carbon superlattices as carbon tends to form different allotropes easily and in the same regions, resulting in an unusual microstructure.

We assume that the barrier potential in the *sp*^3^-*C* regions takes the form of a Gaussian distribution due to the imperfect nature of the interface between the *sp*^3^-*C* and *sp*^2^-*C* regions^20^ (this potential is represented in yellow on the schematic diagram of the superlattice). This broad barrier, without a sharp interface, results in a wide range of resonant energies and spreads out the transmission maxima, in turn resulting in wide resonant features in the calculated I-V characteristics. We assume a relatively small difference in the potential energy between the *sp*^2^-*C* and *sp*^3^-*C* regions in an attempt to reproduce the experimental data. We find prominent NDR at potential differences of around 0.25 *V* and 0.31 *V*. These low potential differences are consistent with the measured I-V properties.

The inset in Fig. 2b shows the calculated I-V characteristics of a superlattice with 4 barriers of width around 8 *nm* (also following a Gaussian form) and 3 wells with a width of approximately 2 *nm* (much narrower than in the previously considered device). We find prominent step-like features related to resonant transmission but no distinct NDR. A closer match to the experimental data was found by slightly changing the hopping parameter in the *sp*^2^ - *C* regions which would reflect a slight change in the overall ratio of *sp*^2^/*sp*^3^ bonding. These regions of current saturation are consistent with the measured I-V characteristics where the devices have smaller well widths. Examination of Fig. 2b also shows step-like features at higher potentials (around 5 *V* at 87 *K*).

These calculations demonstrate that as the well width decreases, the eigenenergies shift to higher potentials however the relatively strong disorder suppresses NDR. Numerous calculations considering different well widths (without changing the barrier height) show that the steps in the I-V characteristics are not very sensitive to the well width while the width of the current steps can be modified by changing the barrier width. The most significant factor by far is the barrier height, with even relatively small changes in the barrier height resulting in dramatic suppression of the current in the low bias region. Calculations of the I-V characteristics for the device shown in Fig. 2a (not shown here) are very similar to the inset in Fig. 2b which is consistent with the experimental data as the current suppression is quite similar for both devices.

**Figure 3:**
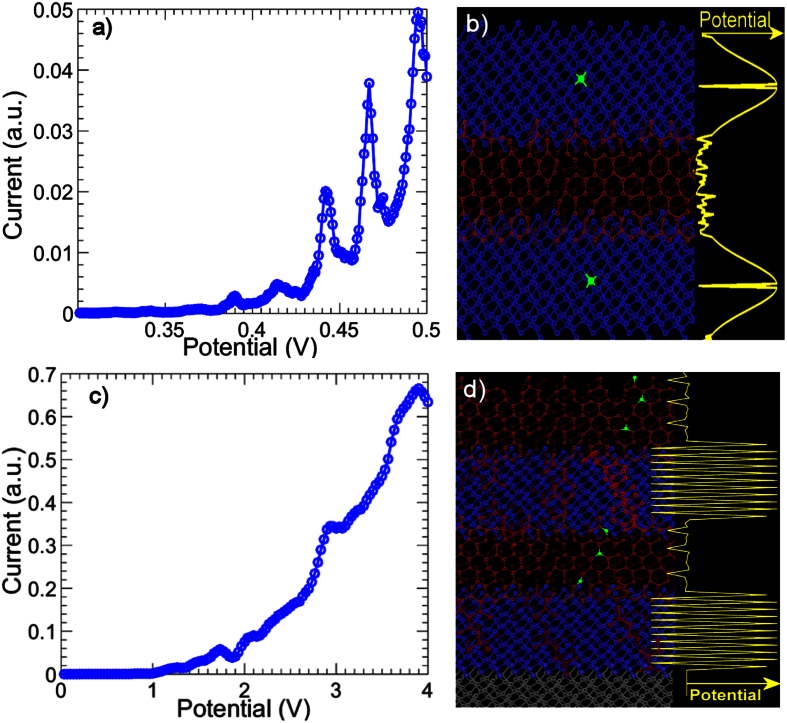
(**a**) Calculated current-voltage characteristics of a diamond-like carbon superlattice with 4 barriers of 7 nm and 3 well of 7 nm with nitrogen trap states incorporated in the *sp*^3^-*C* regions. (**b**) Schematic of the potential for quantum wells and barriers with nitrogen incorporated substitutionally in the *sp*^3^-*C* regions (green impurities). (**c**) Calculated current-voltage characteristics of a DLC superlattice with 4 barriers of 7 nm and 3 well of 7 nm assuming significant bond alternation in the *sp*^3^-*C* regions. (**d**) Schematic showing bond alternation in the *sp*^3^-*C* regions.

We now present some calculations on modified superlattices to illustrate further scope for tuning the quantum transport properties. Figure 3(a) shows the I-V characteristics calculated for a superlattice with 4 barriers and 3 wells both 7 *nm* in length (comparable to Fig. 1a,b) with nitrogen impurities incorporated in the *sp*^3^-*C* regions. Figure 3(b) shows a schematic of the device configuration as well as the potential. These nitrogen impurities create trap states and illustrate how resonant transmission, and consequently the regions of NDR, can be tuned by the inclusion of nitrogen impurities which is of significance for future device applications. The inclusion of nitrogen trap states shifts the eigenenergies to lower energies where the superlattice is less transparent. This results in sharper resonances despite the inherent disorder, resulting in more prominent and sharply defined regions of NDR in comparison to devices with no nitrogen inclusion. The resulting sharp signatures could point the way to quantum devices suitable for quantum information processing.

Figure 3(c) shows the calculated current voltage characteristics for a device with the same dimensions as in Fig. 3(a) but with significant bond alternation in the *sp*^3^-*C* regions (with the same level of disorder in the *sp*^2^-*C* regions). Regions of NDR move to higher potential differences as the bond alternation shifts the eigenenergies to the band edges. This also increases the difference in potentials at which NDR occurs. Prominent NDR is found at 1.8 *V* and close to 4 *V* while less prominent NDR occurs at around 3 *V*. Figure 3(d) shows a schematic of the potential in the *sp*^3^-*C* regions. Bond-alternation is assumed to occur through polymeric chains (shown in red) which spread through the *sp*^3^ hybridized regions due to the affinity of carbon to form chain-like structures^21^.

## Discussion

In the high field regime, we fit the I-V data using Poole-Frenkel emission, Schottky emission and space charge limited conduction models to examine the charge transfer through the structures. Considering the microstructure of these devices, these effects can be expected to play a role in the high field regime^22^,^23^. Only one device though (device with 3 wells of width 2 *nm* and 4 barriers of width 8 *nm*) shows a reasonable fit to the Poole-Frenkel emission model in the high field regime (shown in Supplementary Fig. 1). Fits for the other devices are not convincing for either Poole-Frenkel or Schottky emission or space charge limited conduction. This is likely due to a combined contribution from all these effects which can be expected due to the inherent disorder in the material.

Devices with relatively thicker barriers than in previous studies^12^ (7–8 *nm*) that decoupled wavefunctions within each well structure have been studied to demonstrate the viability of DLC superlattices. The calculated I-V characteristics verify the features of the measured I-V characteristics on a theoretical basis. The differences between the calculations and the measurements can generally be ascribed to the relatively high temperatures of the measurements and the likelihood of Poole-Frenkel/Schottky emission and space charge effects, which are not taken into account in the calculations. The calculations clearly show that the current steps (and small regions of NDR) in the measurements are a result of resonant transmission in the DLC superlattices. In addition, they show that the most prominent effect of disorder is to broaden features in the I-V characteristics. Calculations considering different configurations for DLC superlattices clearly show that the barrier height (relative difference between the potential in the *sp*^2^ and *sp*^3^ hybridized regions) has a far greater influence on the I-V characteristics than any other parameter, which is not necessarily the case for crystalline superlattices. The calculated I-V characteristics of a superlattice with barriers of width 7 *nm* and wells of width 1 *nm* is included in Supplementary Fig. 2 to illustrate this. Thus we can infer that the principle difference between the measured samples is the effect of the extent of the *sp*^2^/*sp*^3^ hybridized regions on the barrier height at the interface. This is fundamentally different from conventional superlattices and is essential to take into account for future work in band engineering of these DLC devices as well as other superlattice structures based on disordered materials such as polymers and amorphous films.

An additional consideration is the effect of significant bond alternation in the *sp*^3^ hybridized regions. Nitrogen doped a-C superlattices have been successfully modeled through inclusion of this effect^11^. In the current experiments, the regions of NDR are found at relatively low potentials. This indicates that bond-alternation is not a prominent feature in the present devices as bond alternation shifts resonant energies close to the band edges, consequently separating out the regions of NDR. The differences between the present DLC devices and other DLC superlattices^11^ is likely due to the differences in the synthesis methods (see, for example^24^) which provides further scope for tuning the device properties.

In addition, prominent current-induced hysteresis was found in these devices. This suggests that conducting channels were formed in the superlattice structures at high bias potentials. Beyond having applications as memory devices, this effect indicates that much of the conduction is through channels within the superlattice and can give rise to a new material to be considered for quantum information processing.

## Conclusion

Quantum confinement effects are demonstrated through current suppression and NDR in DLC superlattices. These temperature dependent features in the measured I-V characteristics are interpreted through calculations of the I-V characteristics using a 1*D* model for structurally disordered superlattices within the Landauer-Büttiker formalism. The calculations indicate that the extent of the *sp*^2^/*sp*^3^ hybridized regions results in changes of the potential barrier at the interface. This effect, far less prominent in crystalline superlattices, has a strong influence on features in the current-voltage characteristics and can be used to inform the design of future devices.

## Methods

### Model

Due to the high affinity of carbon structures to form bonds with different configurations, DLC superlattices are inherently structurally disordered in the form of non-uniform bond lengths within the superlattice structure (schematically shown in Fig. 1b). The bond length can be directly related to the hopping integral through a bond-length deformation potential^18^,^21^. We therefore model the effects of structural disorder through a Gaussian distribution of the hopping integral about some mean value. We consider a tight-binding model of a quasi 1*D* disordered carbon superlattice given by


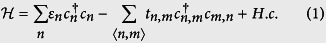


where *ε*_*n*_ is the on-site energy, 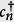 (*c*_*n*_) is the Fermion creation (annihilation) operator and *t*_*n*,*m*_ is the hopping integral where hopping is considered over nearest neighbours. We then solve the discretized Schrödinger equation at each site given by


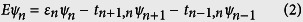


To determine the transmission coefficient, we determine the incoming wave amplitude *A* and define the transmission coefficient as 
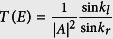
, where *k*_*r*_ is the wave vector of the outgoing plane wave at the right lead and *k*_*l*_ is the wave vector of the plane wave incident on the left lead. The transmission coefficient is then integrated using the Landauer-Büttiker formula to calculate the current-voltage (I-V) characteristics


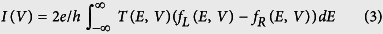


*f*_*L*(*R*)_ is the Fermi function of the left (right) lead, *e* is the electron charge and *h* is Plank’s constant.

In the *sp*^3^-*C* regions, we also consider the possibility of bond alternation (schematically shown on Fig. 3(d)). This is a common feature in conjugated polymers and carbon networks^11^ and results in significant variation of the hopping integral between adjacent lattice sites^21^ due to significant bond angle distortions. Figure 3(d) also shows a schematic of polymer chains embedded in predominantly *sp*^3^-*C* regions, forming links across *sp*^2^-*C* regions.

### Device Fabrication and Measurements

DLC superlattice structures were grown on a highly doped Si substrate by ablation of a rotating graphite target using a 248 *nm* pulsed ultraviolet excimer laser (Lambda Physik LPX 210i) with 25 *ns* pulses at a chamber pressure of 10^−7^ mbar. Alternating layers of predominantly *sp*^3^ hybridized carbon and layers with a smaller *sp*^3^-*C* percentage were achieved by varying the laser fluence from 20 *J*/*cm*^2^ to 4 *J*/*cm*^2^ respectively. The alternating layers show significantly different bandgaps (85% *sp*^3^, 2.8 *eV* barrier layer of DLC) and 50% *sp*^3^, 1.5 *eV* (well layer)). Comprehensive structural characterization, determination of the *sp*^2^-*C* content and the bandgap in the alternating layers through plasmon energy loss spectroscopic profiling has already been published^25^,^26^,^27^. We have included a TEM image of a DLC superlattice in Supplementary Fig. 3 with barriers of width 6 *nm* and wells of width 3 nm.

The superlattices were grown on highly conductive n-type Si (111) 6 *cm* away from the graphite target forming the bottom contact while the top contact was made by depositing a 100 *nm* thick gold layer. The error in the thickness of each layer has a maximum variation of 1 *nm*. The transport properties were measured in the two-probe configuration from 77 *K* using a Janis free flow cryogenic micromanipulated probe station with an Agilent B1500A semiconductor device analyzer.

## Additional Information

**How to cite this article**: McIntosh, R. *et al.* Coherent quantum transport features in carbon superlattice structures. *Sci. Rep.* **6**, 35526; doi: 10.1038/srep35526 (2016).

## Supplementary Material

Supplementary Information
