# Supplementary material for: Coherent quantum transport features in carbon superlattice structures
Source: Sci Rep. 2016 Oct 19;6:35526. doi: 10.1038/srep35526 (PMC5069478; doi:10.1038/srep35526)
Supplement: Supplementary Information [file srep35526-s1.pdf]

Supplementary information: Coherent quantum transport features in carbon superlattice structures

R. McIntosh[1], S. J. Henley[2], S. R. P. Silva[2,*], and S. Bhattacharyya[1,*]

[1]Nano-scale Transport Physics Laboratory, School of Physics, and Centre of Excellence in Strong Materials, University of the Witwatersrand, Private Bag 3, WITS 2050, Johannesburg, South Africa
[2]Advanced Technology Institute, University of Surrey, Guildford, Surrey, GU2 7XH, UK
[*]s.silva@surrey.ac.uk
[*]somnath.bhattacharyya@wits.ac.za

ABSTRACT

Supplementary information on the high field transport properties of DLC superlattices, additional calculations of the I-V characteristics which illustrate that the barrier height has a far more drastic effect on resonant transmission that the well width and an additional TEM image of a DLC quantum well structure.

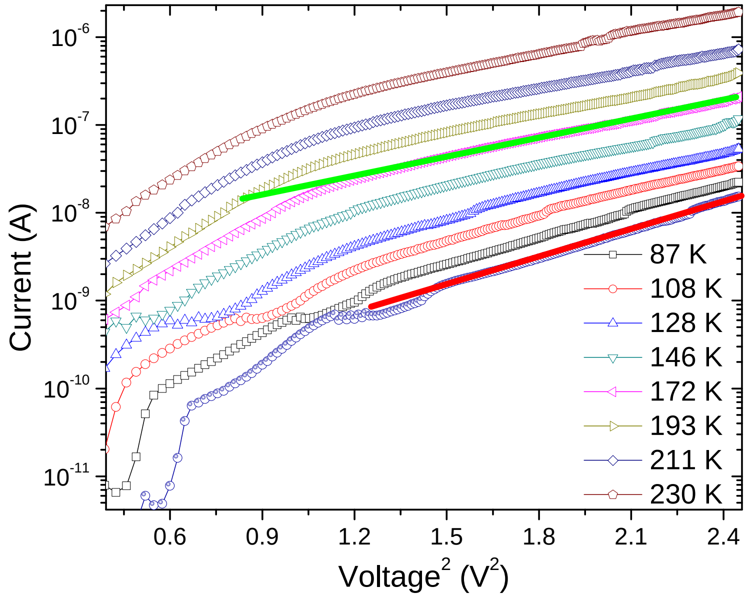

Figure 1. Current as a function of the square root of voltage for a DLC superlattice with 4 barriers of 8 nm and 3 wells of width 2 nm. The red and green lines are linear guides to the eye.

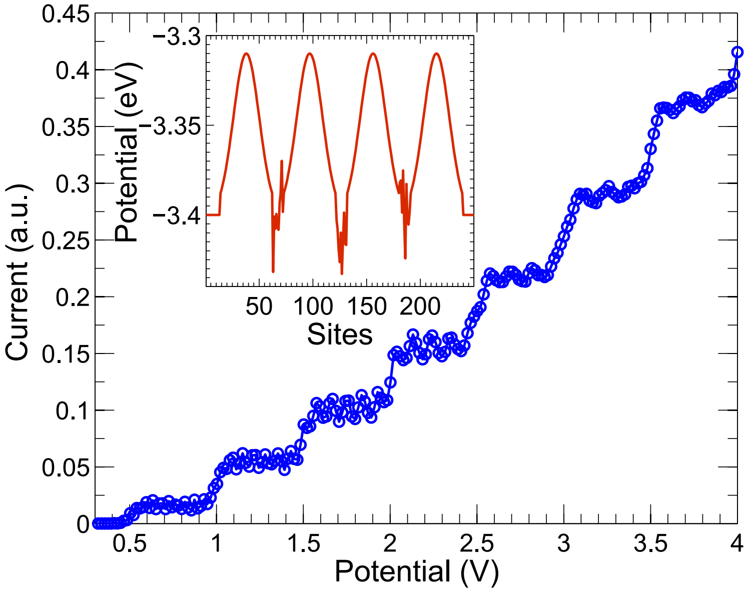

Figure 2. Calculated current-voltage characteristics of a DLC superlattice with 4 barriers of 7 nm and 3 wells of 1 nm assuming a Gaussian distribution of around a mean hopping parameter in the sp^3 - C regions.

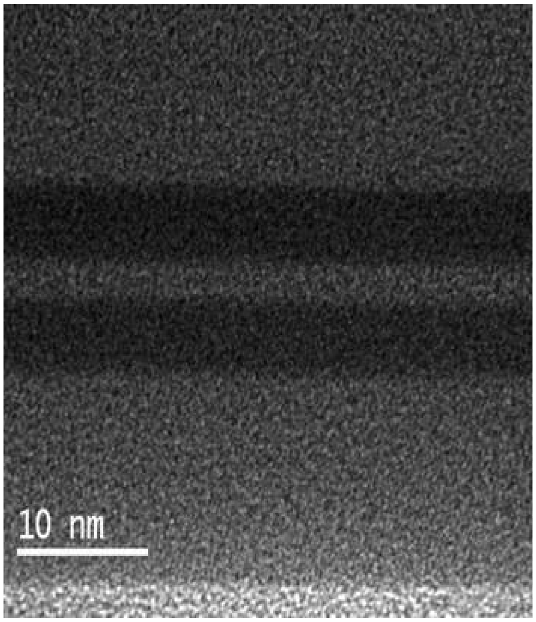

Figure 3. TEM image of a DLC quantum well structure with barriers of width 6 nm and a wells width of 3 nm.
